# Age-related reduction of antibody response against the human endogenous retrovirus K envelope in women

**DOI:** 10.18632/oncotarget.7307

**Published:** 2016-02-10

**Authors:** Hyoung Jin Kim, Byung-In Moon, Jun Woo Lee, Seung Cheol Kim, Hong-Jin Kim

**Affiliations:** ^1^ Laboratory of Virology, College of Pharmacy, Chung-Ang University, Dongjak-Gu, Seoul 06974, South Korea; ^2^ Breast and Thyroid Cancer Center, Ewha Womans University College of Medicine, Yangcheon-Gu, Seoul 07985, South Korea; ^3^ Department of Obstetrics and Gynecology, Ewha Womans University College of Medicine, Yangcheon-Gu, Seoul 07985, South Korea

**Keywords:** human endogenous retrovirus K, envelope, breast cancer, cervical cancer, age, Gerotarget

## Abstract

In the present study, the correlation between the antibody response against human endogenous retrovirus K (HERV-K) envelope and human age was investigated. Antibody levels were compared in groups in their 20s (n = 25), 30s (n = 39), 40s (n = 68), 50s (n = 32), and 60s and over (n = 25), which included healthy individuals and breast cancer and/or cervical cancer patients. It appeared that both IgM and IgG responses against the HERV-K envelope fell with increasing age. There were no differences in anti-HERV-K envelope antibody levels between healthy individuals and cancer patients. Therefore, our results indicated that the anti-HERV-K antibody levels cannot be considered as cancer-specific marker. Also, IgG1 appeared to be the predominant subtype in the reduction of the IgG response by age. Receiver operating characteristic curves of anti-HERV-K envelope IgM levels indicated that the groups of people in their 20s or 30s could be distinguished from those in their 40s, 50s or 60s and over with satisfactory sensitivity and specificity. These findings indicate that the serum antibody level of HERV-K envelope is a critical parameter reflecting person's age.

## INTRODUCTION

The genomes of the human endogenous retrovirus (HERV) occupy about 8% of our genome [1]. It appears that HERVs infected the ancient human germ line, and its genes were integrated into chromosomes and transferred to offspring more than a million years ago [1]. HERVs are a subclass of transposable elements, which constitute an enormous part (about 45%) of the genome sequence of modern humans and have the ability to be packaged and integrated into the genome to accomplish roles in gene expression [2, 3]. In many cases, the genes of HERVs are found to contain deletions, to lack stop codons and to be frame-shifted, leading to loss of their inherent functions. Genetic fragments originating from HERV genomes have been found not only in humans but also other vertebrates; they have been amplified by repeated events of integration and have spread throughout the genome during evolution [4].

Some of the HERVs that were recently integrated into our genome continue to code for proviruses and have open reading frames (ORFs) encoding functional viral polypeptides. HERV-K (HML-2) is the most recent entrant and has been intensively studied because its genome is well-preserved and it is involved in various diseases [5]. HERV-Ks are thought to have been integrated 150,000 years ago, and make up less than 1% of the human genome [6]. Previously, several research groups have suggested that elevated levels of HERV-K transcripts are found in malignant tissues such as germ cell tumors, prostate cancers, ovarian cancers, melanomas and breast cancers [7-11]. Positive roles of HERV genes are also well known. Syncytin-1 and -2 derived from HERV-W and HERV-FRD, which are expressed in the placenta, induce local immunological tolerance that prevents rejection of the fetus [1]. It has also been suggested that expression of HERV-K envelope proteins in placental cytotrophoblast cells is involved in placentogenesis and pregnancy [12]. Knockout of syncytin-A, corresponding to the human syncytin gene, in the mouse resulted in disruption of placental architecture and death of the embryos [13]. It has also been proposed that HERV gene fragments are permanent components of the human transcriptome, since HERV transcripts are present in most human tissues [14]. These positive and negative roles of HERVs raise the fundamental questions whether HERVs act as autoantigens or exogenous pathogens.

It is clear that HERVs do not cause complete immunological tolerance [15]. In fact expressed HERV antigens provide B-cell and T-cell epitopes recognized by the human immune system [15]. Previous studies have suggested that enhanced antibody responses against HERV-K are associated with carcinogenesis in germ cells, prostate and breast [10, 11, 16]. On the other hand, others have reported that there were no differences in anti-HERV antibody levels between healthy donors and patients with leukemia, brain tumors, mammary cancers or multiple sclerosis [17]. Also, there is evidence that antibodies against HERV-K are found in healthy donors [18]. Taken together, these previous reports indicate that antibody responses against HERV-K are not limited to disease states and raise the possibility that unknown factors are involved in antibody responses against HERV-K.

At this point, investigations of the immune responses to HERV-K antigens from different points of view are needed to extend our knowledge of their roles. Age is undoubtedly a risk factor for cancer and for many other diseases. In the present study, we investigated the correlation between antibody responses to HERV-K envelope protein (env) and age.

## RESULTS

### Purification of GST-HERV-K env su protein

**Figure 1:**
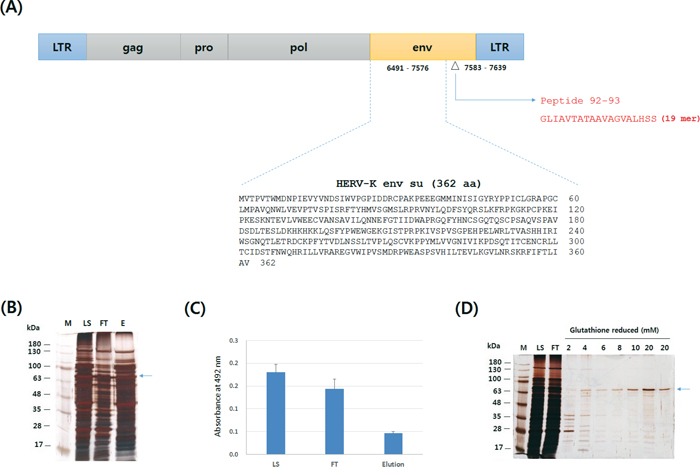
Schematic diagram showing the amino-acid sequences of HERV-K env su and the peptide used in this study, and the purification of GST-HERV-K env su proteins The amino acid sequences of HERV-K env su (362 amino acids) and peptide 92 – 93 are shown in panel **A.** 6491 – 7576 and 7583 – 7639 gene residues on HERV-K precursor genome (GeneBank accession no.: M14123.1) encode 362 amino acids of HERV-K env su and 19 amino acids of peptide 92 – 93, respectively. The GST-HERV-K env su protein was purified by DEAE chromatography **B.** followed by GST affinity chromatography **D.** Panel **C.** presents the result of the ELISA showing the levels of GST-HERV-K env su protein in the DEAE chromatography fractions. ELISA data are means ± standard errors of the means (SEMs) of triplicates. The proteins in panels B and D were visualized by silver staining. Arrows indicate the locations of GST-HERV-K env su protein. SDS-PAGE images were acquired by Window 7 paint program.

The env surface unit (su) of HERV-K was expressed as a GST-fusion protein, as described previously with modifications [19]. Previously, peptide 92 – 93 residue of HERV-K env was suggested as B-cell and T-cell epitope [11]. The amino acid sequences of HERV-K env su and of peptide 92 – 93 are shown in Figure 1A. We first tried single-step GST-affinity chromatography to purify the GST-HERV-K env su protein. However, it could not be purified by a single step of GST-affinity chromatography; many kinds of contaminating proteins were found in the elution fractions of the GST-affinity chromatography (data not shown). Therefore, GST-HERV-K env su protein was first enriched by DEAE chromatography to remove contaminating proteins, followed by GST-affinity chromatography. As shown in Figure 1B, the flow-through fraction of DEAE chromatography contained only a small amount of protein compared to the elution fraction and the flow-through fraction contained a significantly higher amount of the GST-HERV-K env su protein than the elution fraction (Figure 1C). Therefore, the flow-through fraction was collected and further purified by GST-affinity chromatography. Figure 1D shows that the env su protein was recovered with satisfactory purity after GST-affinity chromatography.

### Comparison of anti-HERV-K env su antibody levels in normal, breast cancer and cervical cancer groups

**Figure 2:**
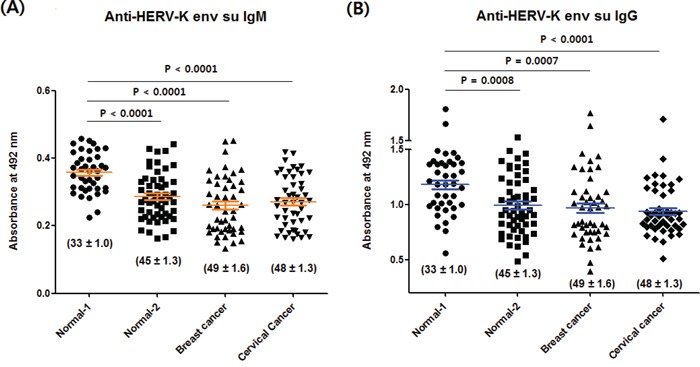
Anti-HERV-K env su IgM and IgG levels of the normal-1, normal-2, breast cancer and cervical cancer group Anti-HERV-K env su IgM **A.** and IgG **B.** levels were measured as described in MATERIALS AND METHODS. Numbers in parenthesis refer to the means ± SEMs of age. The values of individual sera are represented with dots. The central line through the dots represents the mean, and the top and bottom whiskers show the SEMs. Normal-1, n = 40; normal-2, n = 54; breast cancer, n = 47; cervical cancer, n = 48.

Two types of normal groups were designed to compare anti-HERV-K env su antibody levels. Normal-1 group (mean age: 33 ± 1.0) consisted of women not diagnosed with breast cancer while normal-2 group (mean age: 45 ± 1.3) consisted of women not diagnosed with cervical cancer (Table 1). Since the mean ages of the breast cancer and cervical cancer groups were 49 ± 1.6 and 48 ± 1.3, respectively (Table 1) normal-1 group was considerably younger than the other groups. There were no differences in anti-HERV-K env su IgM (Figure 2A) and IgG (Figure 2B) levels between the normal-2, breast cancer and cervical cancer groups whereas the IgM (Figure 2A) and IgG (Figure 2B) levels of normal-1 group were significantly higher than those of the normal-2, breast cancer or cervical cancer groups. Therefore, only age-related differences were found in these experimental sets.

**Table 1:** Age distribution of each group

Group	Number of person						Age (Mean ± SEM)
	20s	30s	40s	50s	60s and over	Sum	
Normal-1^a^	16	20	3	1	–	40	33 ± 1.0
Normal-2^b^	4	7	29	9	5	54	45 ± 1.3
Breast cancer^c^	–	6 Stage I (n = 3) Stage II (n = 2) Stage III (n = 1)	21 Stage 0 (n = 2) Stage I (n = 9) Stage II (n = 9) Stage III (n = 1)	11 Stage I (n = 4) Stage II (n = 4) Stage III (n = 3)	9 Stage I (n = 5) Stage II (n = 4)	47	49 ± 1.6
Cervical cancer^d^	5 Stage I (n = 5)	6 Stage I (n = 5) Stage II (n = 1)	15 Stage I (n = 15)	11 Stage I (n = 11)	11 Stage I (n = 8) Stage II (n = 2) Stage III (n = 1)	48	48 ± 1.3
Sum	25	39	68	32	25	189	44 ± 0.9

a diagnosed without breast cancer

b diagnosed without cervical cancer

c determined by TNM staging

d determined by FIGO staging

### Comparison of anti-HERV-K env su antibody levels in different age groups

**Figure 3:**
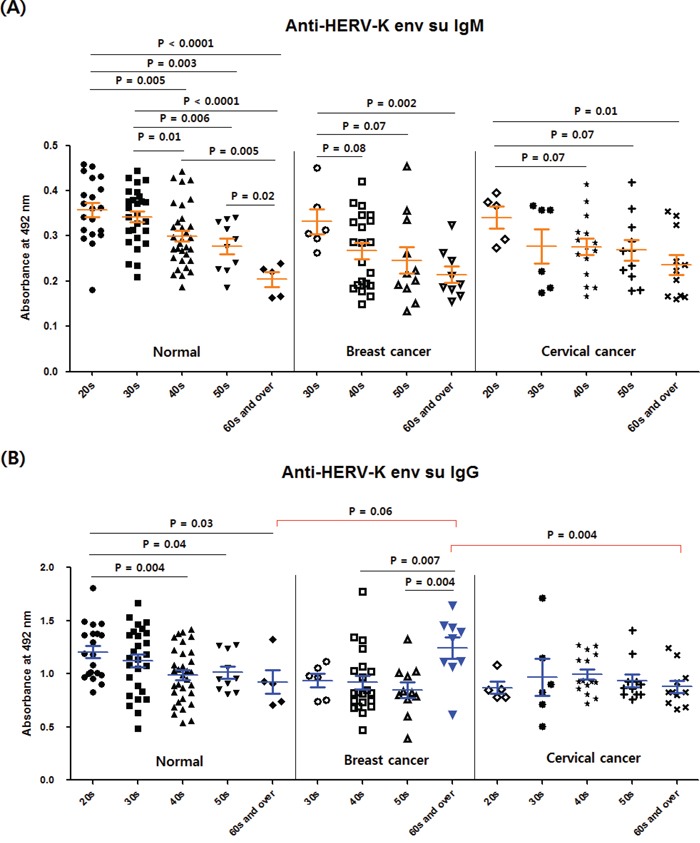
Comparison of anti-HERV-K env su IgM and IgG levels between different age groups in the normal, breast cancer and cervical cancer groups The normal, breast cancer and cervical cancer groups were arranged according to age, and the anti-HERV-K env su IgM **A.** and IgG **B.** values were compared between groups. The normal group contains the normal-1 and 2 groups. The numbers of individuals in the age subgroups within the normal group were as follows: 20s, n = 20; 30s, n = 27; 40s, n = 32; 50s, n = 10; 60s and over, n = 5. The numbers of individuals in the age subgroups within the breast cancer group were as follows: 30s, n = 6; 40s, n = 21; 50s, n = 11; 60s and over, n = 9. The numbers of individuals in the age subgroups within the cervical cancer group were as follows: 20s, n = 5; 30s, n = 6; 40s, n = 15; 50s, n = 11; 60s and over, n = 11. Other details are as for Figure 2.

Figure 3 shows the anti-HERV-K env su IgM (A) and IgG (B) levels in the normal, breast cancer and cervical cancer groups arranged according to age. The IgM levels fell with increasing age in the normal, breast cancer and cervical cancer groups (Figure 3A), whereas a declining trend in IgG level was found only in the normal group (Figure 3B). Meanwhile, the 60s and over subgroup of the breast cancer group had a significantly higher IgG level than not only the 40s and 50s subgroups of the breast cancer group (P < 0.01) but also than the age-matched cervical cancer group (P < 0.01) (Figure 3B). Also the IgG value of the 60s and over subgroup of the breast cancer group was higher than that of the age-matched normal group (P = 0.06) (Figure 3B). As shown in Table 1 there was no biased-cancer stage in the 60s and over group of the breast cancer, compared to other age groups of the breast cancer group.

**Figure 4:**
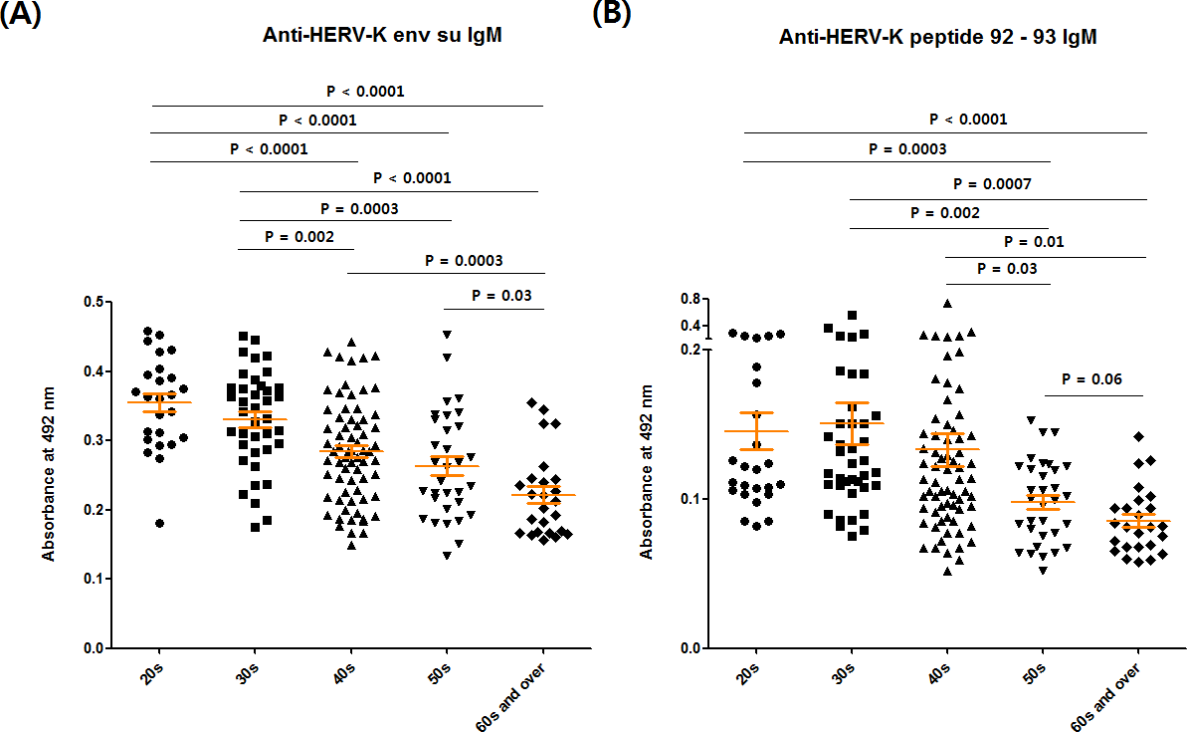
Comparison of anti-HERV-K env su IgM and anti-HERV-K env peptide 92 – 93 IgM levels between different age groups The values of anti-HERV-K env su IgM **A.** and anti-HERV-K env peptide 92 – 93 IgM **B.** for all groups (normal-1, normal-2, breast cancer and cervical cancer) were combined and arranged according to age. The numbers of individuals in the age subgroups were as follows: 20s, n = 25; 30s, n = 39; 40s, n = 68; 50s, n = 32; 60s and over, n = 25. Other details are as for Figure 2.

Comprehensive results for the values of anti-HERV-K env su IgM in the different age groups that included all values of the normal, breast cancer and cervical cancer groups, showed that the IgM response against HERV-K env su always fell with age (Figure 4A). Similar results were obtained for the anti-HERV-K env peptide 92 – 93 IgM response (Figure 4B).

**Figure 5:**
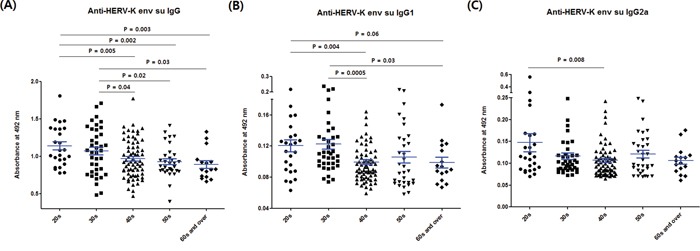
Comparison of anti-HERV-K env su IgG, IgG1 and IgG2a values between different age groups The values of anti-HERV-K env su IgG, IgG1 and IgG2a are presented in panels **A, B.** and **C.** respectively. The 60s and over in the breast cancer group (n = 6) were excluded from the 60s and over group. The numbers of individuals in the age subgroups were as follows: 20s, n = 25; 30s, n = 39; 40s, n = 68; 50s, n = 32; 60s and over, n = 19. Other details are as for Figure 4.

The reduction in anti-HERV-K env su IgG response by aging was clearer in the comprehensive comparison between age groups containing all values of the normal, breast cancer and cervical cancer groups (Figure 5A): the value for the 60s and over subgroup of the breast cancer group was excluded due to its exceptional antibody response. The results for anti-HERV-K env su IgG1 (Figure 5B) and IgG2a (Figure 5C) indicate that the decline of the anti-HERV-K env su IgG response with age is biased towards the IgG1 subclass. The IgG1-biased reduction was also seen when the normal group alone was analyzed (Supplementary Figure S1). Therefore, it seems that the Th2-like immune response against HERV-K env is involved in the reduction of IgG response with age.

**Figure 6:**
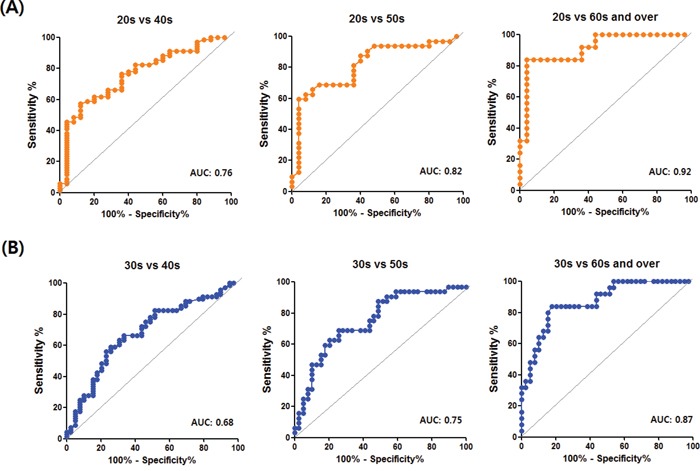
ROC curves discriminating the 20s or 30s from the 40s, 50s, 60s and over in the ELISAs detecting anti-HERV-K env su IgM ROC curves discriminating the 20s from the 40s, 50s or 60s and over and discriminating the 30s from 40s, 50s or 60s and over are presented in **A.** and **B.** respectively.

The power values (1-β) of the comparison of the different age groups indicate that the decreasing trend of the antibody response with age is statistically reliable (Table 2). Moreover, the differences between the age groups in anti-HERV-K env su IgM level were sufficient to discriminate the 20s or 30s groups from the 40s, 50s or 60s and over groups with satisfactory area under the curve (AUC) values, indicating that the IgM level can be used as a parameter for estimating age (Figure 6). Taken together, these results demonstrate that there is a reduction in antibody responses against HERV-K env that is significantly linked to age, and that the IgM response is predominant in the age-related reduction of the antibody response.

**Table 2:** Evaluations of statistical significance and power (1-β) values in comparison of age groups. Red bolds indicate P < 0.05 (statistical significance) and power values over 0.8

Comparison	Anti-HERVK-env su IgM		Anti-HERVK-env peptide 92-93 IgM		Anti-HERVK-env su IgG	
	P value	Power (1-β error)	P value	Power (1-β error)	P value	Power (1-β error)
20s vs 30s	0.18	0.75	0.80	0.55	0.38	0.68
20s vs 40s	**< 0.0001**	**0.98**	0.53	0.63	**0.005**	**0.92**
20s vs 50s	**< 0.0001**	**0.99**	**0.0003**	**0.96**	**0.002**	**0.94**
20s vs 60s and over	**< 0.0001**	**0.99**	**< 0.0001**	**0.98**	**0.003^a^**	**0.94^a^**
30s vs 40s	**0.002**	**0.94**	0.34	0.68	**0.04**	**0.83**
30s vs 50s	**0.0003**	**0.97**	**0.002**	**0.95**	**0.02**	**0.88**
30s vs 60s and over	**< 0.0001**	**0.99**	**0.0007**	**0.97**	**0.03^a^**	**0.88^a^**
40s vs 50s	0.19	0.73	**0.03**	**0.89**	0.45	0.68
40s vs 60s	**0.0003**	**0.97**	**0.01**	**0.93**	0.27	0.73
50s vs 60s and over	**0.03**	**0.86**	0.06	**0.83**	0.57	0.62^a^

a Breast cancer group was excepted from 60s and over group.

## DISCUSSION

### Comparison of our results and previous results for antibody responses against HERV-K in healthy donors and patients with cancer

Unlike the clear correlation between the expression level of HERV-K and cancer progression, the correlation between antibody response against HERV-K and cancer progression remains uncertain. A recent study suggested that elevated levels of anti-HERV-K envelope antibodies in American women were associated with breast cancer development and could be used as a serologic biomarker to screen breast cancer [11]. However, another study did not find the enhanced antibody levels of anti-HERV-K GAG in breast cancer patients, compared to normals, in groups made up of combinations of American and German women [10]. Hervé et al reported that the level of anti-HERV-K envelope IgG was high in both healthy British women and those with autoimmune rheumatic disease [18]. In the case of Korean women, in the present study, there were no differences in age-matched comparisons between the normal group and the breast cancer and cervical cancer groups, except for the 60s and over breast cancer group (Figure 3B). It has been suggested that the geographic distribution of HERV-K genes varies [20]. Our results and previous results imply that the antibody response against HERV-K may depend on ethnicity and/or geographic location.

### The declining trend in anti-HERV-K env antibody level with age found in the present study differs from the general trend of autoantibody level with age

We found that not only the IgM but also IgG levels of anti-HERV-K env fell with increasing age. Previously, Nagele and colleagues suggested that levels of IgG autoantibodies rise with increasing age [21]. Mouthon et al. found that serum IgM autoantibodies are acquired in early childhood and remain constant throughout life [22]. Gonzalez-Quintela et al. suggested that total serum IgM was not affected by age while total serum IgA and IgG increased with age [23]. Therefore, the declining patterns for IgM and IgG against HERV-K env seen in this study are different from the general trends of serum antibodies and autoantibodies as a function of age. Previous reports indicate that HERVs have dual properties of autoantigens and exogenous pathogens [5, 24, 25]. Therefore it may be that both these aspects of HERV-K antigens make difference in the patterns of antibodies with age.

### The IgG1 subtype is predominant in the reduction of anti-HERV-K IgG response with age

IgG1 and IgG2a are derived from Th2 and Th1 type immune responses, respectively. Th1 type immune responses are primarily mediated by intracellular antigens while Th2 type immune responses are primarily mediated by soluble antigens [26]. We found that IgG1 is the predominant subtype in the reduction of the anti-HERV-K IgG response with age (Figure 5B and Supplementary Figure S1). Various human fluids has been thought to contain considerable levels of elements derived from HERVs: for example, HERV-K env mRNAs and retrovirus-like particles in the blood of patients with cancer [27] and retrovirus-like particles in the milk of healthy donors [28, 29]. Therefore it is likely that the IgG response against HERV-K env is mainly mediated by soluble antigens derived from HERV-K.

### The importance of age matching in investigations of antibody responses to HERV-K

Previous reports yielded conflicting results for the antibody response against HERV-K in patients with autoimmune diseases. One study suggested that elevated levels of antibodies reacting with HERV-K env peptides were present in systemic lupus erythematosus (SLE) patients [18]. However, another study failed to find any difference in levels of antibodies against HERV-K env peptides between healthy donors and patients with SLE [30].

Rates of type 2 diabetes and RA are associated with age [31]. The incidence of RA peaks in the 70 – 79 year age group [32] while 90% of SLE occurs in fertile young women [33]. This age difference in the incidence rates of different autoimmune diseases implies that there are age-biased factors implicated in these diseases. Our results indicate that antibody responses against HERV-K env are high in women in their 20s and 30s (Figure 4 and 5). Therefore, age-matching between diseased and healthy individuals may be important in future studies.

### A possible explanation for the elevated levels of anti-HERV-K env antibodies in younger women

HERV env proteins have immunosuppressive properties similar to those of HIV env [34], and it has been suggested that HERV env can promote tumor development via its immunosuppressive function [1, 35]. Cancer incidence is related to age, and this effect is linked to reduced immune function with age [36]. Therefore, we suggest that the elevated levels of IgM and IgG antibodies against HERV-K env su in younger women may increase resistance against the immunosuppressive activities of HERV-K.

## MATERIALS AND METHODS

### Ethics

This study was conducted with the approval of the Ewha Womans University Mokdong Hospital Institutional Review Board (approval No. EUMC 2014-11-013-001 and ECT 13-15A-28). The research was conducted in accordance with the Declaration of Helsinki. Patient samples were obtained after written informed consents.

### Specimens

Samples were collected in a prospective and consecutive manner. Sera from women diagnosed without breast cancer (normal-1 group, n = 40) and with breast cancer (n = 47) were collected from the Breast and Thyroid Cancer Center. Sera from women diagnosed without cervical cancer (normal-2 group, n = 54) and with cervical cancer (n = 48) were collected from the Department of Obstetrics and Gynecology. Breast and cervical cancer stages were determined by the TNM and FIGO staging system, respectively, and the corresponding sera were stored at −80°C.

### Preparation of recombinant HERV-K env su

The HERV-K env su protein was expressed as a GST-fusion protein, as described previously with modifications [19]. The 1086 bp sequence encoding HERV-K env su was synthesized by Bioneer (South Korea) and ligated into pGEX 4T-1 using the EcoR I and Not I sites to produce glutathione S transferase (GST)-fusion protein (GST-HERV-K env su). BL21 *E. coli* cells were transformed with the pGEX 4T-1 vector (GE Healthcare, USA) harboring the HERV-K env su gene, and expression of GST-HERVK env su was induced by isopropyl-β-D-thio-galactoside (IPTG, final conc. 1 mM) at 28°C for 4 h.

Cultured cells (100 ml culture) were disrupted by sonication for 10 min in cell disruption buffer (10 mM sodium phosphate dibasic, 50 mM NaCl, 1.7 mM EDTA, 1 mM PMSF, 0.1% β-mercaptoethanol, 1% Triton X-100, pH 10.2) and clarified by centrifugation at 12,000 *g* for 10 min. GST-HERV-K env su protein in the supernatant was loaded onto DEAE CL-6B resin (2 ml, GE Healthcare, USA) equilibrated with the above cell disruption buffer, and the flow-through fraction was collected.

The pH of flow-through fraction of DEAE chromatography was adjusted to 6.8 by addition of potassium phosphate monobasic. The sample was loaded onto glutathione Sepharose 4B resin (0.5 ml, GE Healthcare, USA) equilibrated with binding buffer (10 mM sodium phosphate dibasic, 0.15 M NaCl, 0.1% β-meraptoethanol, 1% Triton X-100 pH 6.8). After three successive loadings, the resin was washed with 7 column volumes of the binding buffer. Thereafter, GST-HERV-K env su proteins bound to the resin were eluted by addition of elution buffer containing successively 2, 4, 6, 8, 10 and 20 mM reduced glutathione (1 ml each, Sigma, USA). The reduced glutathione was prepared in buffer (50 mM Tris pH 9.2, 0.15 M NaCl, 0.02% Triton X-100, 0.1% β-mercaptoethanol). The GST-HERV-K env su was fractionated on 12% polyacrylamide gel and visualized by silver staining to confirm its purity.

### Enzyme-linked immunosorbent assay for detecting GST-HERV-K env su

An enzyme-linked immunosorbent assay (ELISA) was performed to monitor the amounts of GST-HERV-K env su protein contained in the loading sample, flow-through fraction and elution fraction of the DEAE chromatography. The wells of a 96-well microplate (Greiner, Germany) were coated with the various fractions diluted 1:10 with phosphate-buffered saline (PBS) and blocked with 5% skim milk in PBS containing 0.05% Tween 20 (PBS-T). The GST-HERV-K env su protein was then detected with mouse anti-GST serum obtained from a GST-immunized mouse, together with horse radish peroxidase (HRP)-conjugated anti-mouse IgG (Bethyl, USA). Three washes were carried out between reactions. Color reactions were developed with *o*-phenylenediamine (Sigma, USA) and measured at 492 nm.

### Preparation of HERV-K env peptide 92 - 93

Peptides 92–93 (GLIAVTATAAVAGVALHSS, 19-mer) of the HERV-K envelope [11] was synthesized as a multiple antigen peptide, that is, a branched peptide, by PEPTRON (South Korea).

### ELISA for measuring serum anti-HERV-K env su and peptide 92-93 antibodies

96-well microplates (Greiner) were coated overnight with 50 ng/well of purified GST-HERV-K env su protein in PBS or 2 μg/well of HERV-K env peptide 92-93 in 75% trifluoroacetic acid (Sigma) at 4°C and blocked with 5% skim milk in PBS-T. The plate reacted with human serum, which was diluted 1:50 or 1:300 with 0.5% skim milk in PBS-T, for 2 h at 37°C.

The anti-HERV-K env su IgG, IgG1 and IgG2a were detected using anti-human IgG (Sigma A8667, USA), anti-human IgG1 (PIERCE, MH1715, USA) and anti-human IgG2a (PIERCE, MH1722) antibodies, respectively. Similarly, the anti-HERV-K env su IgM and anti-HERV-K env peptide 92-93 IgM were detected using anti-human IgM antibody (Sigma, A6907). All secondary antibodies were conjugated with HRP. Three washes were carried out between reactions. The development of color reactions and measurement of optical densities (OD) were performed as described above.

### Statistical analysis

Differences between groups were analyzed using two-tailed Student's t-tests. *P* < 0.05 was considered statistically significant. Statistical power values (1-β error) were calculated using the G*power 3.1 program. ROC curves and area under the curve (AUC) values were obtained using GraphPad Prism 5.01.

## SUPPLEMENTARY FIGURE


