# Supplementary material for: Age-related reduction of antibody response against the human endogenous retrovirus K envelope in women
Source: Oncotarget. 2016 Feb 10;7(14):17327–37. doi: 10.18632/oncotarget.7307 (PMC4951215; doi:10.18632/oncotarget.7307)
Supplement: Supplementary file 1 [file oncotarget-07-17327-s001.pdf]

SUPPLEMENTARY FIGURE

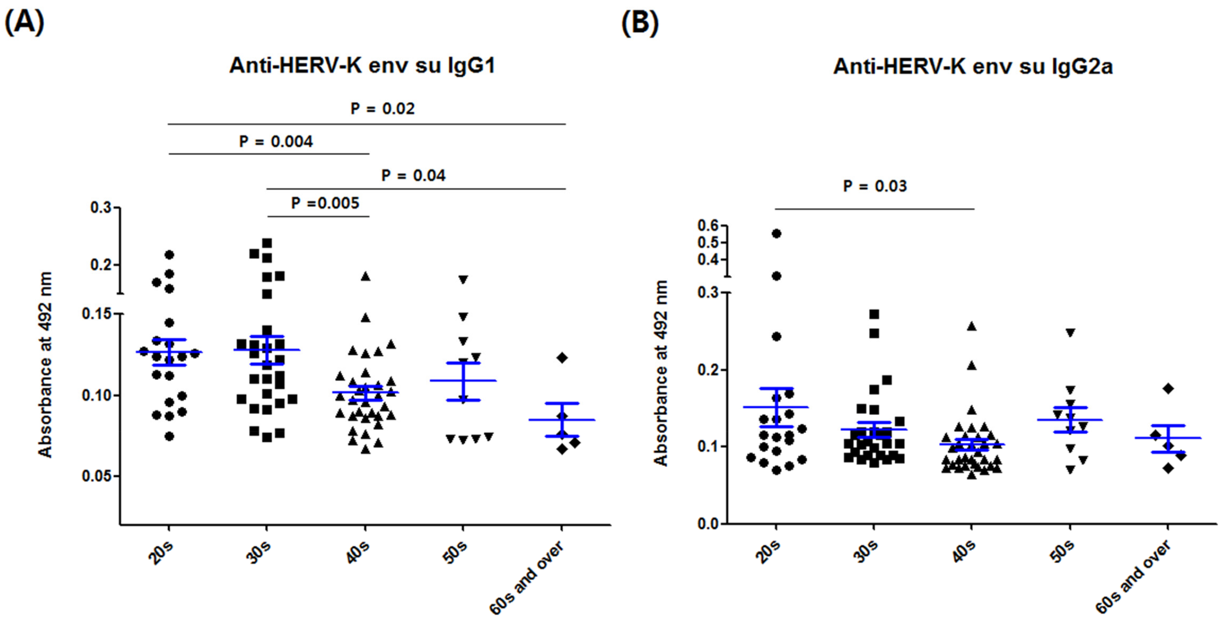

Supplementary Figure S1: Comparison of anti-HERV-K env su IgG1 and IgG2a values in the different age groups in the normal group. The values of anti-HERV-K env su IgG1 and IgG2a are presented in **A.** and **B.** respectively. The numbers in the age subgroups of the normal group were as follows: 20s, n = 20; 30s, n = 27; 40s, n = 32; 50s, n = 10; 60s and over, n = 5.
